# Crystal fingerprinting: elucidating the crystals of Cheddar, Parmigiano-Reggiano, Gouda, and soft washed-rind cheeses using powder x-ray diffractometry

**DOI:** 10.1007/s13594-015-0225-6

**Published:** 2015-05-08

**Authors:** G. F. Tansman, P. S. Kindstedt, J. M. Hughes

**Affiliations:** Department of Nutrition and Food Sciences, University of Vermont, Burlington, VT USA; Department of Geology, University of Vermont, Burlington, VT USA

**Keywords:** X-ray diffraction, Crystals, Cheese, Tyrosine, Leucine

## Abstract

Crystals in cheese may be considered defects or positive features, depending on the variety and mode of production (industrial, artisanal). Powder x-ray diffractometry (PXRD) offers a simple means to identify and resolve complex combinations of crystals that contribute to cheese characteristics. The objective of the present research was to demonstrate the application of PXRD to study crystals from a range of different cheese types, specifically Cheddar, Parmigiano-Reggiano, Gouda, and soft washed-rind (smear ripened) cheeses. In studies of Parmigiano-Reggiano and long-aged Gouda, PXRD has confirmed that hard (crunchy) crystals that form abundantly within these cheeses consist of tyrosine. Furthermore, PXRD has tentatively identified the presence of an unusual form of crystalline leucine in large (up to 6 mm in diameter) spherical entities, or “pearls”, that occur abundantly in 2-year-old Parmigiano Reggiano and long-aged Gouda cheeses, and on the surface of rindless hard Italian-type cheese. Ongoing investigations into the nature of these “pearls” are providing new insight into the roles that crystals play in the visual appearance and texture of long-aged cheeses. Crystals also sometimes develop profusely in the eyes of long-aged Gouda, which have been shown by PXRD to consist of tyrosine and the aforementioned presumptive form of crystalline leucine. Finally, crystals have been shown by PXRD to form in the smears of soft washed-rind cheeses. These crystals may be associated in some cheeses with gritty mouth feel and with zonal body softening that occurs during ripening. Heightened interest in artisanal cheeses highlights the need to better understand crystals and their contributions to cheese characteristics.

## Introduction

Crystals that form in cheese have been the subject of scientific inquiry for over a century, and x-ray diffraction (XRD) has been marshaled as a direct analytical approach to identify cheese crystals for almost as long, the earliest studies dating back to the 1930s (Tuckey and Ruehe 1938; Tuckey et al. 1938). Most XRD investigations of cheese crystals have been restricted to Cheddar cheese and have focused primarily on calcium lactate pentahydrate (CLP) crystals (Agarwal et al. 2006; Chou et al. 2003; Conchie et al. 1960; Dybing et al. 1988; Harper et al. 1953; Tuckey et al. 1938; Washam et al. 1985). A few studies of Cheddar also identified the occurrence of crystals consisting of tyrosine (Conchie et al. 1960; Harper et al. 1953; Shock et al. 1948), cysteine (Harper et al. 1953; Shock et al. 1948), and calcium phosphate (Conchie and Sutherland 1965). In the United States, visible crystals are often viewed by the Cheddar industry as defects and sources of confusion for consumers (Johnson 2014). Therefore, there has been considerable interest to understand the factors that promote crystal formation and develop measures to prevent their occurrence. In contrast, in some traditional cheeses such as Parmigiano-Reggiano and hand-crafted artisanal cheeses, crystals are viewed as important contributors to cheese character and quality (Noël et al. 1996; Zannoni et al. 1994). Growing consumer appreciation of traditional and artisanal cheeses is also fueling interest in understanding the nature and origins of crystals and their contributions to cheese quality and character.

Studies of crystals in cheeses other than Cheddar, such as tyrosine crystals in Roquefort (Dox 1911), CLP, calcium phosphate and tyrosine crystals in Grana cheeses (Bottazzi et al. 1982, 1994), calcium phosphate crystals in soft white mold surface-ripened (bloomy rind) cheeses (Boutrou et al. 1999; Brooker 1987; Gaucheron et al. 1999; Karahadrian and Lindsay 1987), and CLP and calcium phosphate crystals in Serra cheese (Parker et al. 1998), have generally employed indirect methods such as chemical analyses or microscopy coupled with differential staining and x-ray microanalysis to identify presumed crystalline species. Although indirect approaches may provide useful presumptive identification of crystalline species, only XRD can furnish direct and definitive results because the XRD pattern of a specific crystal is determined by its three-dimensional atomic arrangement, which is unique to that crystal and thus analogous to a fingerprint. Consequently, the XRD pattern of an unknown species can be compared to those of known crystals; a perfect match with one of the known patterns provides definitive identification. Today, a growing database of over 250,000 known XRD patterns is accessible through the International Centre for Diffraction Data (ICDD).

Powder x-ray diffractometry (PXRD), which utilizes crystalline specimens that ideally have been pulverized to a fine powder, is a versatile and user-friendly application of XRD. Major enhancements in the computing power of PXRD instrumentation, and advances in the software algorithms for data analysis, have opened up new opportunities for the study of cheese crystals. For example, the authors recently demonstrated that the two different enantiomeric forms of CLP that were presumed to occur in Cheddar cheese (i.e., the L(+) and D(−)/L(+) forms) can be identified and differentiated using PXRD, based on subtle differences in the XRD patterns of the two enantiomeric configurations (Tansman et al. 2014). The application of PXRD to study crystals in Cheddar and other cheese varieties could offer new insights into the factors that govern crystallization phenomena in cheese and the roles that crystals play in determining cheese character and quality. Therefore, the objective of the present research was to demonstrate the application of PXRD to study crystals from a range of different cheese types, specifically Cheddar, Parmigiano-Reggiano, Gouda, and soft washed-rind (smear ripened) cheeses.

## Materials and methods

### Cheese samples and crystal collection

All cheese samples were commercially produced and purchased from local retail outlets, and crystals were harvested for analysis as described below, except for Cheddar cheese and a hard Italian-style cheese. In the case of Cheddar, the manufacturer harvested large (up to ca. 5 mm) internal crystals from the body of a Cheddar cheese sample (aged 2 years before sale) and delivered the crystals to the University of Vermont for identification after the manufacturer received a consumer complaint about unknown objects in their cheese. Crystals from a hard Italian-style cheese (obtained courtesy of Mark Johnson, Center for Dairy Research, University of Wisconsin) were scraped from the cheese surface using a spatula. Small (ca. 1–2 mm) dense crystals embedded in the body of Parmigiano-Reggiano cheese samples (aged 2 years) and Gouda cheese samples (aged 2 years) were physically excised from the surrounding cheese matrix using a dissecting needle and tweezers. The isolated crystals were brushed free of adhering cheese matrix. Parmigiano-Reggiano samples also contained white spherical entities that ranged from barely visible to around 6 mm in diameter, which will be referred to as “pearls” in this publication. Pearls were separated from the surrounding cheese matrix by slicing the sample with a wire cutter into 1-cm sections and gently applying pressure to the cheese surrounding the pearl using thumb and index finger. Gentle pressure caused the surrounding matrix to fracture and separate from the pearls, enabling the pearls to be isolated and brushed free of adhering cheese matrix. Similar pearls, though smaller in size distribution, also were harvested from the Gouda samples. Gouda samples also displayed extensive crystal formation along the surfaces of internal eyes. Crystals were scraped free from the surfaces of eyes using a spatula. The six soft washed-rind (smear ripened) cheeses that were examined in this study were produced by different companies, four located in the United States and two in Italy.

### Analytical methods

Pearls collected from Parmigiano-Reggiano samples were evaluated for size distribution by placing them on a vibrating stack of four sieves of successively smaller mesh size (5.6, 4.00, 3.35, and 2.00 mm) and counting the number of pearls retained by each sieve. The pearls were then pooled to form a composite sample, grated to fine particles in a blender, and analyzed in duplicate for total solids content by drying in a forced-draft oven at 100 °C for 24 h, fat content by a modified Babcock method (Atherton and Newlander 1977), and for soluble leucine (i.e., free amino acid) content by a modified gas chromatography–mass spectrometry method as follows. Thirteen milligrams of pearls was homogenized in 100 mL of deionized water adjusted to a pH of 1 with HCl. The suspension was vortexed for 10 min and centrifuged at 5000 rpm for an additional 5 min to remove particulate material from the solution. A 1-mL aliquot of the mixture was removed for analysis and to this was added 0.1 mL of 8.37 mM 555 d_3_ leucine (Isotec 486825 Lot TPI633-SP). The sample was mixed with 1 mL of 50% acetic acid and passed through cation exchange resin (Biorad AG50W-x8 cation exchange resin 100–200 mesh). The resin was then rinsed three times with 2 mL of deionized water. Amino acids were eluted off the resin by the addition of 2 mL of 4 N ammonium hydroxide. The eluate was collected and evaporated under nitrogen gas. To the dry vial was added 200 µL of a 50:50 mixture of *N*-methyl-*N*-(*t*-butyldimethylsilyl) trifluoro-acetamide + 1% *t*-butyldimethylchlorosilane (MtBSTFA + 1% t-BDMCS)/acetonitrile. The solution was heated for 30 min in a heating block at 80 °C and then transferred to an autosampler vial. A 1-µL aliquot was injected into a ZB1 gas chromatographer (GC) column (30 m in length; 0.25 mm inner diameter with 0.25 μm film). The GC was run isothermally at 165 °C with a column flow rate of 1 mL helium per minute. The mass spectrometer (MS) was run in selected ion monitoring mode and monitored for a mass to charge of 200 *m*/*z* for leucine and 203 *m*/*z* for labeled leucine.

Pooled samples of the cheese matrix surrounding the pearls were similarly grated and analyzed for total solids and fat contents. Gouda samples that displayed extensive crystal formation along the surfaces of internal eyes were examined by stereoscopic microscopy before the crystals were harvested for analysis. Smear material was scraped from the surface of soft washed-rind (smear ripened) cheeses employing a spatula, using care not to remove the underlying cheese matrix along with the smear. Smear material was analyzed for selected mineral content by inductively coupled atomic emission spectrometry as follows. A scraping of surface smear from one Vermont washed-rind cheese was dried and defatted in acetone and analyzed for Ca, Mg, K, and P by ICP-OES (Optima 3000DV; Perkin Elmer Corp, Norwalk, CT, USA). Calibration standards were prepared according to the instrument manufacturer’s suggested guidelines, to cover the range of concentrations in the sample set. Two-point calibrations (plus a Calibration Blank) were used for ICP analysis. Continuing Calibration Verification samples, prepared from an independent source, were used to check the calibration periodically.

In preparation for PXRD, dense internal crystals obtained from Cheddar, Parmigiano-Reggiano, and Gouda cheeses, as well as crystals scraped from the surface of the hard Italian-style cheese and from the eyes of Gouda, were dried and defatted in acetone, finely ground using a mortar and pestle, and transferred to a glass diffraction slide with a well. The powder in the slide well was leveled with the surface of the diffraction slide using a microscope slide. Diffraction patterns were generated from cheese crystals using a MiniFlex II powder x-ray diffractometer (Rigaku, The Woodlands, TX). Diffractograms were generated at a speed of 2° 2*θ*.min^−1^ between 5 and 50° 2*θ*. Diffraction patterns were compared to existing entries archived in the ICDD database to determine matches using a proprietary calculation called a “figure of merit”. The figure of merit was calculated automatically using PDXL, the diffractometer software package (Rigaku, The Woodlands, TX). An acceptance threshold of 1.0 was set for the figure of merit. Diffraction patterns were also generated from finely grated samples of pearls, and samples of the surrounding cheese matrix, from Parmigiano-Reggiano and Gouda cheeses as described. Samples of smears scraped from the surfaces of soft washed-rind (smear ripened) cheeses were loaded directly onto the slide well and immediately analyzed without any additional sample preparation.

## Results and discussion

### Cheddar

**Fig. 1:**
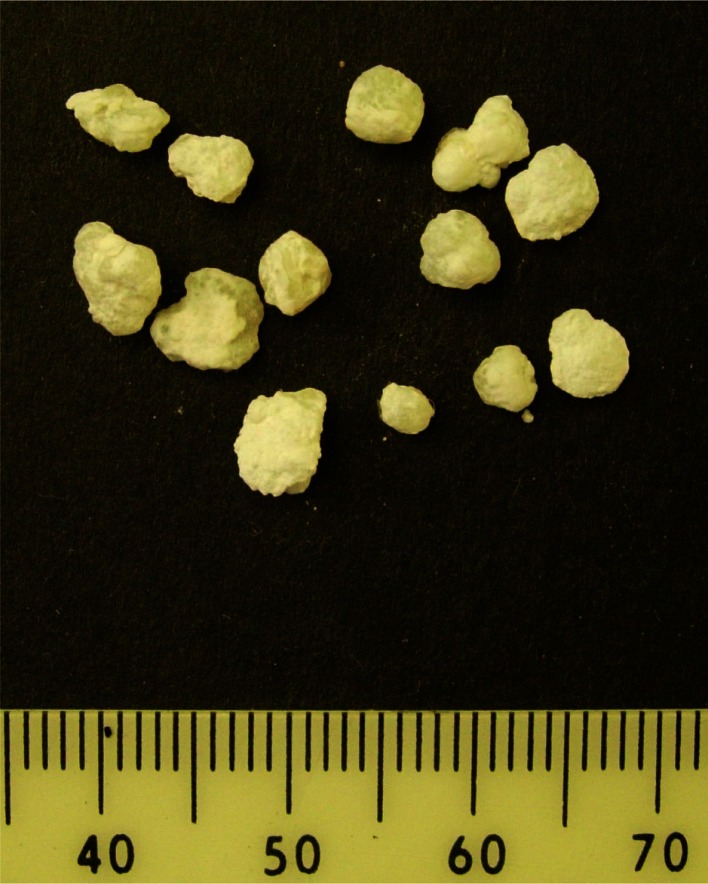
Large interior crystals collected from the body of a commercial Cheddar cheese that was aged for 2 years before distribution for retail sale. The scale is in millimeters

**Fig. 2:**
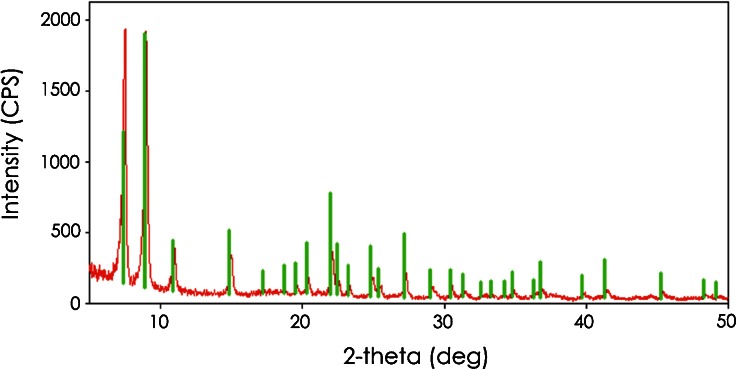
X-ray diffraction pattern (*in red*) from large interior crystals collected from the body of a commercial Cheddar cheese that was aged for 2 years before distribution for retail sale (see Fig. 1). The *green bars* represent the reference card (ICDD card number 00-029-1596) labeled “calcium lactate pentahydrate”

Cheddar cheese crystals in this study were large internal crystals (Fig. 1), not surface crystals, which were of particular interest because their presence triggered a consumer complaint to the cheese manufacturer about possible foreign objects in the cheese. The x-ray diffraction pattern, shown in Fig. 2, identified the crystals as CLP in the D(−)/L(+) enantiomeric configuration based on different XRD patterns of the two enantiomeric forms reported previously (Tansman et al. 2014). CLP crystals at the surface of Cheddar cheese have been studied extensively. However, little work has been conducted on internal crystals, and only one previous report identified large internal CLP crystals as occurring in the D(−)/L(+) form (Tansman et al. 2014). In the present study, the large internal D(−)/L(+) CLP crystals suggest a microbial origin involving nonstarter lactic acid bacteria (NSLAB) because conventional starter cultures for Cheddar cheese are comprised of strains of *Lactococcus lactis* ssp. *lactis* and *cremoris*, which ferment lactose to L(+) lactate exclusively and which lack the capacity to convert L(+) lactic acid to the D(−) form. In contrast, various NSLAB, including several strains of *Pediococcus pentosaceus* (Thomas and Crow 1983) and *Lactobacillus curvatus* (Somers et al. 2001), are able to racemize L(+) lactate to D(−) lactate and promote D(−)/L(+) CLP crystallization. Furthermore, racemizing NSLAB have been shown to produce biofilms on equipment surfaces, which can be a source of contamination leading to the formation of D(−)/L(+) CLP crystals in Cheddar (Somers et al. 2001). Thus, a possible strategy to reduce or prevent the occurrence of large internal D(−)/L(+) CLP crystals might involve identifying and eradicating the offending source of NSLAB contamination. Alternatively, D(−) lactate in Cheddar cheese could also be produced if *Lactobacillus helveticus*, which ferments lactose to D(−) lactate, were included in the starter culture, as is sometimes practiced in Cheddar manufacture to enhance flavor (Johnson 2014). In this scenario, corrective action might involve removing *Lb. helveticus* from the starter. In either case, determining the enantiomeric form of CLP by PXRD can provide useful diagnostic information.

### Parmigiano-Reggiano

**Fig. 3:**
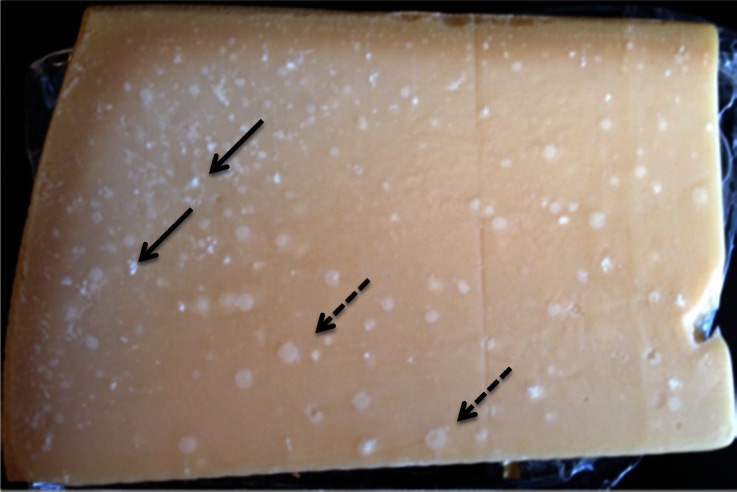
Visual appearance of a wedge sample of commercially produced Parmigiano-Reggiano cheese that was aged for 2 years before retail distribution. Small white crystals (*solid arrows*) and pale white spherical “pearls” (*dashed arrows*) are noted

**Fig. 4:**
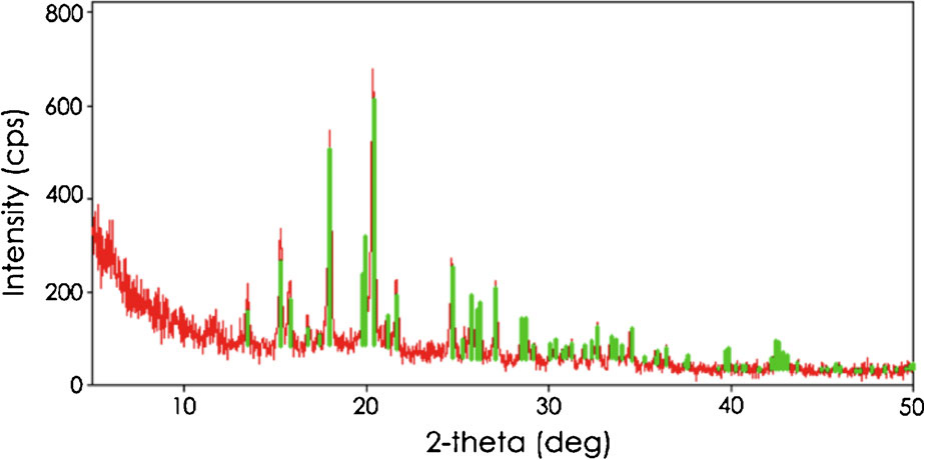
X-ray diffraction pattern (*in red*) from small white interior crystals collected from the body of a commercial Parmigiano-Reggiano cheese that was aged for 2 years before distribution for retail sale (see Fig. 3). The *green bars* represent the reference card (ICDD card number 00-039-1840) labeled “tyrosine”

In traditional practice, Grana-type cheeses such as Parmigiano-Reggiano are pierced and pried open, which results in very rough and irregular internal cheese surfaces. In contrast, Parmigiano-Reggiano cheese for retail sale in the United States is sometimes cut into wedge-shaped slices with very smooth surfaces and then vacuum packaged in clear film. Smooth surfaces accentuate visible heterogeneities within the cheese body, such as small white crystals (Fig. 3, solid arrows) and spherical pearls that appear as pale white circles against the darker cheese matrix (Fig. 3, dashed arrows). Thus, these heterogeneous elements strongly influence the visual appearance of cheese samples packaged in this manner for the American market. Small white entities in Parmigiano-Reggiano (Bottazzi et al. 1982) and Grana Padano (Bianchi et al. 1974) cheese were presumptively identified as tyrosine crystals based on analyses of amino acid content, but studies using XRD to confirm the crystalline identities of such elements are lacking. The diffraction pattern generated by PXRD (Fig. 4) from the small white elements collected from Parmigiano-Reggiano (Fig. 3, solid arrows) confirms that they were tyrosine crystals, and only tyrosine crystals.

**Fig. 5:**
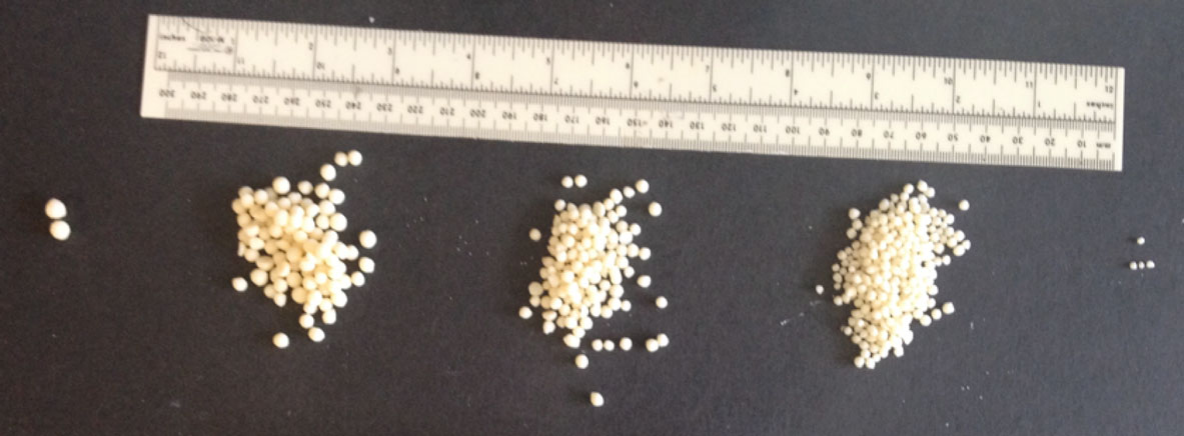
“Pearls” having diameters ≥2 mm, which were collected from a single sample of Parmigiano-Reggiano cheese weighing ca. 680 g. The pearls are grouped according to retention on sieves of successive mesh size. The ruler is 300 mm long

The striking abundance of visible pearls in the Parmigiano-Reggiano samples, which the authors have also observed in similarly packaged samples of Grana Padano, prompted closer examination. Pearls having diameters ≥2 mm, collected from a single cheese sample weighing ca. 680 g, are shown in Fig. 5. The pearls are grouped according to size based on retention by sieves of successive mesh sizes ranging from 5.60 to 2.00 mm. The pearls displayed remarkably consistent near spherical geometries irrespective of size (Fig. 5). Approximately 67 pearls per 100 g of cheese, which accounted for about 2% of the total weight of the cheese, were collected from the sample. The volume fraction of the cheese matrix occupied by pearls in this cheese appeared to be quite considerable. Given the abundance of the pearls, and their distinctly different body relative to that of the cheese matrix, which enabled the pearls to be easily harvested by hand, it seems likely that pearls influence the fracture properties of the cheese. The size distribution of pearls harvested from five different commercial samples of Parmigiano-Reggiano, all weighing about 680 g, is presented in Table 1. All five samples contained a few pearls greater than 5.6 mm in diameter, many more between 4 and 5.6 mm, and the greatest number between 2 and 4 mm. Assuming that all pearls grow at the same rate, the smallest pearls harvested were presumably the most recent to form during aging. Continued aging of the cheese, therefore, would be expected to result in nonlinear increases in the volume fraction occupied by pearls as the large number of smaller pearls increase in diameter. Such a bloom in pearl volume is likely to exert an increasingly greater effect on cheese fracture properties as aging progresses.

**Table 1:** Average number of “pearls” collected from five different commercial samples of Parmigiano-Reggiano cheese

Sieve mesh size	Mean^a^	Standard deviation^a^
5.60	7	8
4.00	127	46
2.00	321	84

Each cheese sample weighed approximately 680 g. Means and standard deviations correspond to the numbers of pearls retained on stacked sieves of decreasing mesh size from 5.60 to 2.00 mm

^a^ *n* = 5

The total solids and fat contents of composite samples of the pearls and surrounding cheese matrix are shown in Table 2. The total solids content was 8.72% higher in the pearls than in the cheese matrix, but the fat content was 4% lower, indicating that the higher solids content of the pearls was not caused simply by greater loss of moisture from the pearls relative to losses from the surrounding matrix during aging. Instead, the accumulation of solids other than fat contributed to higher total solids, and lower fat content, in the pearls relative to the matrix; presumably, this increase in solids was associated with the growth in scaffolding that enabled the pearls to expand spherically, and which contributed to the firmer texture of the pearls relative to the surrounding matrix. The high fat content of the pearls (28%) indicates that pearls’ scaffolding engulfed and occluded fat globules as the pearls increased in diameter.

**Table 2:** Compositional analyses of a composite sample of “pearls”, and the cheese matrix surrounding the pearls, collected from five different commercial samples of Parmigiano-Reggiano cheese

	Pearls	Cheese matrix
Total solids (%)	79.09	70.37
Fat (%)	28	32
FDM (%)	35.4	45.5
Free leucine (%)	1.0	–

**Fig. 6:**
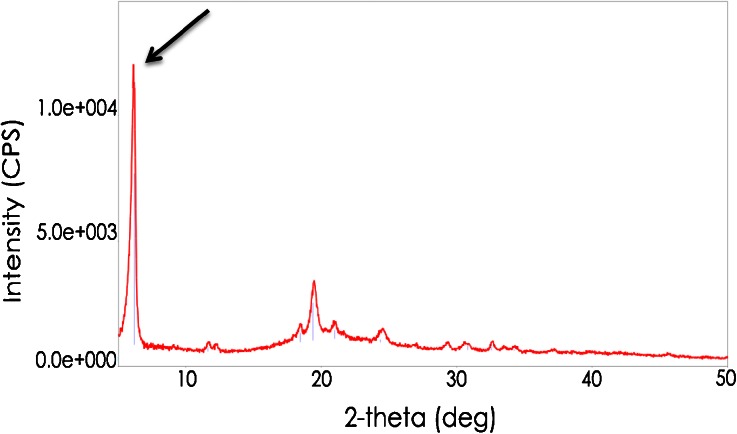
X-ray diffraction pattern from a composite sample of interior “pearls” collected from five commercial Parmigiano-Reggiano cheese samples that were aged for 2 years before distribution for retail sale. The pattern displays a prominent peak at 6.00° 2*θ* (see *arrow*)

**Fig. 7:**
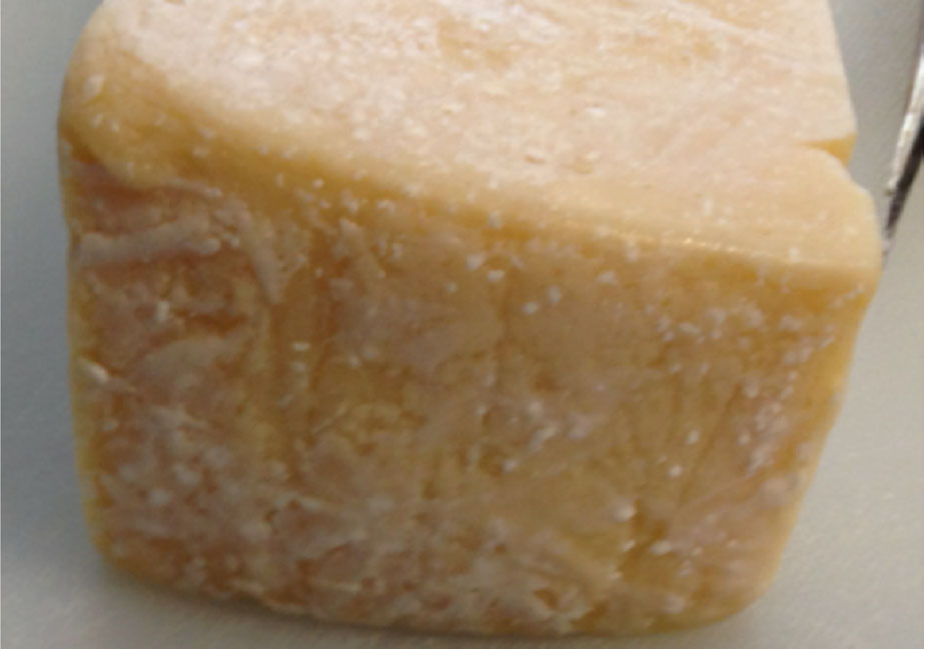
Surface of a hard Italian-style cheese from the United States displaying extensive crystal coverage

The chemical makeup of the pearl scaffolding and its structural organization are unknown but may be related to crystallization phenomena. For example, Bianchi et al. (1974) observed large (up to 6 mm) entities in Grana Padano cheese, apparently similar to the pearls observed in the present study, which they referred to as “spots”. Spots collected from cheeses aged for 18 and 25 months contained around 9.9 and 7.7% leucine, respectively (Bianchi et al. 1974). Such extraordinarily high leucine levels suggest a possible structural role of crystalline leucine in the Grana Padano spots. However, the composite sample of pearls from Parmigiano-Reggiano in the present study contained only 1.0% leucine present as free amino acids. Currently, the authors have no explanation for this discrepancy, but it is worth noting that analysis of the Parmigiano-Reggiano pearls by PXRD gave a diffraction pattern, shown in Fig. 6, that corresponded to a partial diffraction pattern for crystalline leucine, which is characterized by a very strong peak at 6.00° 2*θ*. In contrast, a composite sample of the cheese matrix surrounding the pearls did not diffract x-rays (data not shown). Sou et al. (2013) recently observed a similar partial diffraction pattern for leucine, which they postulated corresponds to an alternative crystalline form of leucine. Further studies will be needed to determine whether this presumptive alternative leucine crystal contributes to the structural scaffolding of pearls in Parmigiano-Reggiano and Grana Padano cheeses. However, it is interesting to note that crystals collected from the surface of the hard Italian-style cheese, shown in Fig. 7, also yielded the same partial diffraction pattern for leucine (data not shown), indicating that the same presumptive crystals can occur in both pearls located internally in cheese and at the surface of cheese when the right conditions are present.

### Gouda

**Fig. 8:**
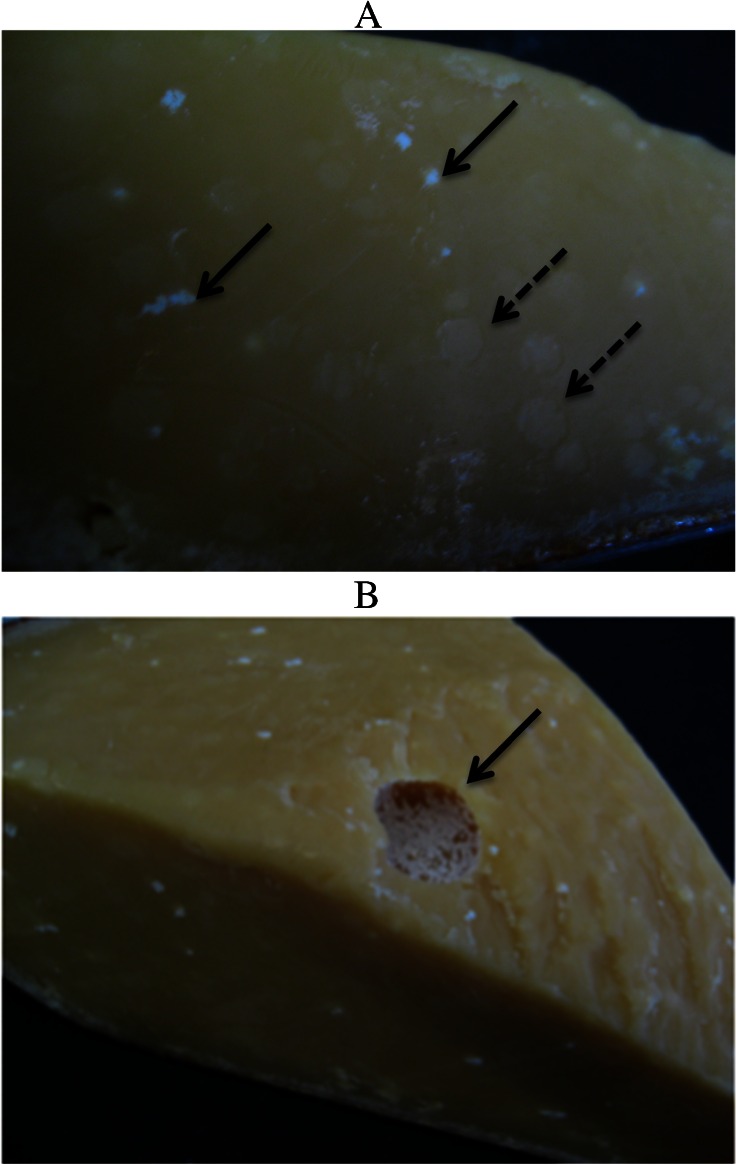
Visual appearance of a commercially produced Gouda cheese that was aged for 2 years before retail distribution. Small white crystals (*solid arrows*, **a**) and pale white spherical “pearls” (*dashed arrows*, **a**) are noted. Also, eyes within the cheese were lined with crystals (*solid arrow*, **b**)

Like Parmigiano-Reggiano, the 2-year aged Gouda samples in this study displayed visible heterogeneities within the cheese body, including small white crystals (Fig. 8a, solid arrows) and spherical pearls that appear as pale white circles against the darker cheese matrix (Fig. 8a, dashed arrows). Profuse crystal blooms also were observed in the eyes of the cheese (Fig. 8b, solid arrow). Analysis by PXRD confirmed that the small white crystals embedded in the cheese body consisted of tyrosine (data not shown), and that the pearls contained the presumptive alternative form of crystalline leucine characterized by an intense XRD peak at 6.00° 2*θ* (data not shown), similar to the pattern obtained with Parmigiano-Reggiano pearls (Fig. 6). This is perhaps surprising because the starter cultures used for Gouda traditionally do not include the thermophilic strains used in Grana type cheeses, although the inclusion of thermophilic adjuncts in Gouda manufacture is becoming more common (Düsterhöft et al. 2011). Again, further studies will be needed to determine whether the presumed crystalline leucine contributes to the structural scaffolding of pearls.

**Fig. 9:**
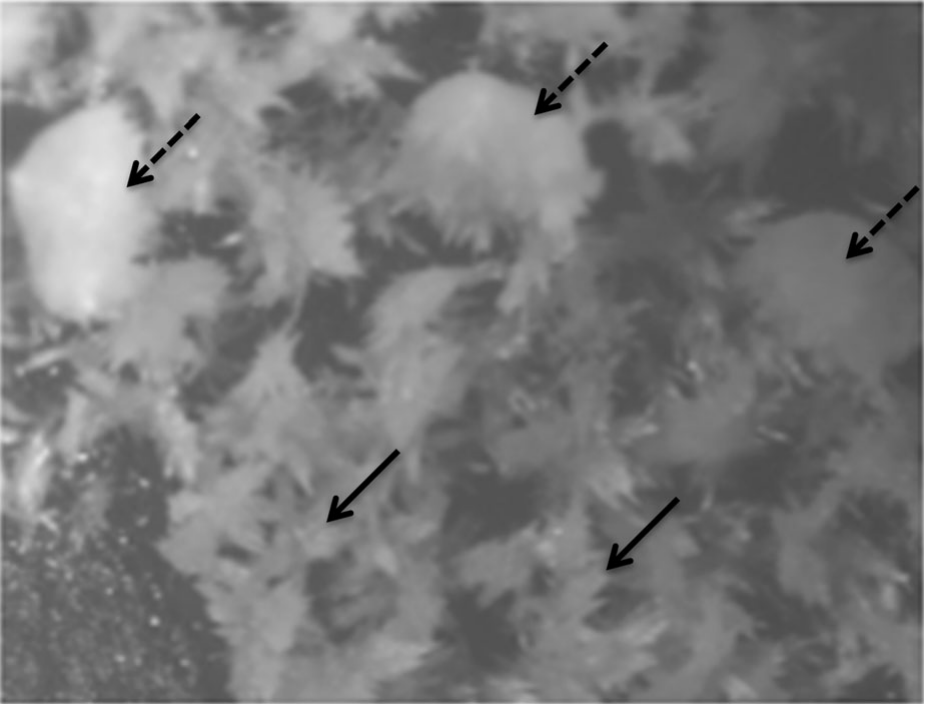
Phase contrast photomicrograph (×11.25 magnification) of crystals lining the surface of an eye in a commercially produced Gouda cheese that was aged for 2 years before retail distribution (see Fig. 8b). Two different crystal morphologies, one characterized by an open structure (*solid arrows*) and the other by a compact structure (*dashed arrows*), appear to be present

**Fig. 10:**
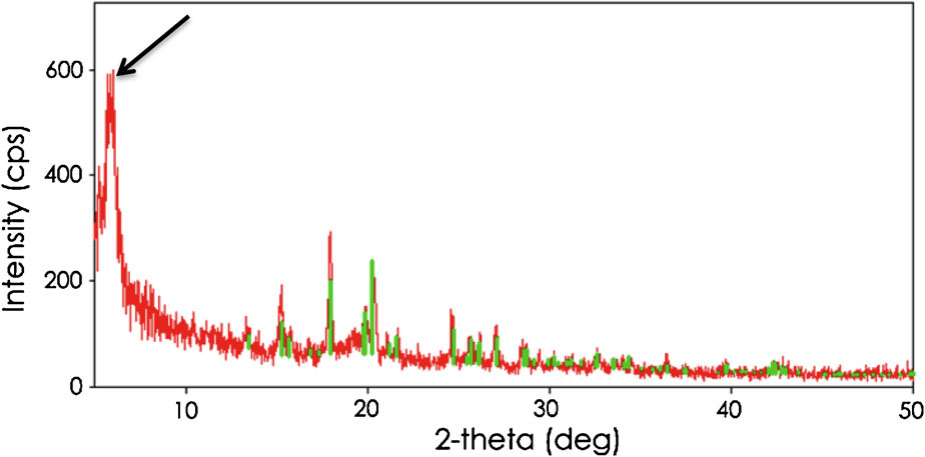
X-ray diffraction pattern (*in red*) from crystals collected from the surfaces of eyes in a commercial Gouda cheese sample that was aged for 2 years before distribution for retail sale (see Fig. 8b). The *green bars* represent the reference card (ICDD card number 00-039-1840) labeled “tyrosine”. Also evident is a prominent peak at 6.00° 2*θ* (see *arrow*)

When the crystals that lined the eyes of Gouda were observed using stereophase contrast microscopy, the visual appearance suggested the presence of two different crystal morphologies, one characterized by an open structure (Fig. 9, solid arrows) and the other by a compact structure (Fig. 9, dashed arrows). The diffraction pattern of the crystals generated by PXRD confirmed the presence of two different crystalline species: tyrosine crystals plus leucine in the presumed alternative configuration, as indicated by the intense peak at 6.00° 2*θ* (Fig. 10). Thus, the same presumptive crystals can occur in both pearls located internally in cheese and on a cheese surface, in this case the surface of eyes.

### Soft washed-rind (smear ripened) varieties

**Fig. 11:**
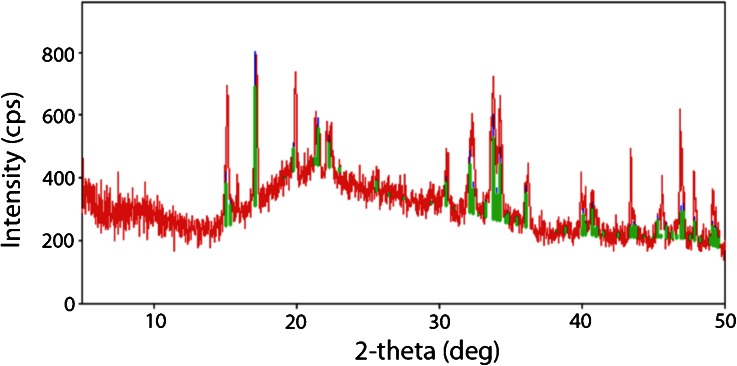
X-ray diffraction pattern (*in red*) from smear material collected from surface of a commercial soft washed-rind cheese. The *green bars* represent the reference card (ICDD card number 01-074-7174) labeled “ikaite”

The mineral content of a surface smear collected from one of the soft washed-rind (smear ripened) cheeses is shown in Table 3. Calcium and P were present in the smear at concentrations around ten times higher than that of other minerals measured, which suggests that calcium and phosphate ions migrated from the cheese body to the surface in a manner similar to what is believed to occur in bloomy rind cheeses, i.e., as a result of high surface pH that develops during ripening, which triggers surface crystallization of calcium phosphate (Boutrou et al. 1999; Brooker 1987; Gaucheron et al. 1999; Karahadrian and Lindsay 1987). Therefore, the authors expected to find one or more forms of crystalline calcium phosphate in the smears of the six soft washed-rind cheeses that were analyzed by PXRD. However, smears from all six cheeses each produced a unique XRD pattern that could not be matched to any pattern in the ICDD database. Diffraction of x-rays indicates the presence of crystals in a sample, thus the XRD patterns confirmed that crystalline species were present in the smears of all six cheeses but none could be identified. A possible explanation for the unknown XRD patterns became evident during the course of the analyses when the authors noted the samples dried out extensively during the diffraction procedure, dehydrating the extant phases. This strongly suggested that the PXRD results contained diffraction artifacts of the experimental conditions. Consequently, the preparation of smear samples for PXRD was modified to include the addition of a thin layer mineral oil over the sample well to prevent sample desiccation. This modification produced reproducible data, thereby preempting artifacts obtained previously. When the modified sample preparation procedure was applied to smear material from two of the washed-rind cheeses analyzed previously, the XRD pattern for ikaite, which consists of calcium carbonate in an unusual hexahydrate form, was identified in the smears (Fig. 11). Additional analyses of other washed-rind cheeses are underway and will be presented in a subsequent report.

**Table 3:** Concentrations (g.kg^−1^) of selected minerals in dried and defatted smear material collected from the surface of a soft washed-rind (smear ripened) cheese

Mineral	Concentration (g.kg^−1^)
Ca	78.66
P	33.26
K	4.34
Mg	2.10
Na	9.67

The formation of surface crystals, whether of calcium phosphate or other species, is of great interest because of the possible implications for zonal softening, which has been studied extensively in bloomy rind cheeses but not well characterized in soft washed-rind cheeses. Surface crystal formation also has implications for the development of sandiness or grittiness, which the authors have heard anecdotally from cheesemakers and cheesemongers may be considered desirable or undesirable depending on the market.

## Conclusion

Powder x-ray diffractometry is a versatile and powerful analytical approach to identify and differentiate crystalline species in cheese. Crystals influence the quality and character of many cheeses, ranging from extra hard, long-aged types, such as grana cheeses, to soft, rapidly ripened cheeses such as surface ripened washed-rind and bloomy-rind types. This study confirmed the presence of tyrosine crystals and a crystalline species presumptively identified as leucine in both Parmigiano-Reggiano and Gouda cheeses, as well as large interior crystals of D(−)/L(+) calcium lactate pentahydrate in Cheddar cheese and ikaite crystals at the surface of a soft washed-rind cheese. Advancements in PXRD technology have facilitated new avenues of research relating to structure-function implications of crystallization phenomena, such as the role of leucine crystals in the formation and growth of pearls in Parmigiano-Reggiano and Gouda cheeses, and the possible role of surface crystals in the zonal softening of soft surface ripened cheeses.
